# Identification of Tumor Antigens and Immune Subtypes of Esophageal Squamous Cell Carcinoma for mRNA Vaccine Development

**DOI:** 10.3389/fgene.2022.853113

**Published:** 2022-06-06

**Authors:** Tong Lu, Ran Xu, Cheng-Hao Wang, Jia-Ying Zhao, Bo Peng, Jun Wang, Lin-You Zhang

**Affiliations:** Department of Thoracic Surgery, The Second Affiliated Hospital of Harbin Medical University, Harbin, China

**Keywords:** esophageal squamous cell carcinoma (ESCC), immune landscape, immune subtype, mRNA vaccine, tumor antigens

## Abstract

**Purpose:** The applicability of mRNA vaccines against esophageal squamous cell carcinoma (ESCC) remains unclear. Here, we identified potential antigens for developing mRNA vaccines against ESCC and characterized immune subtypes to select appropriate patients for vaccination.

**Methods:** RNA-seq, genetic alteration data, and corresponding clinical information of ESCC patients were obtained from the Cancer Genome Atlas (TCGA) database. The RNA-seq data of normal esophageal tissue were obtained from the Genotype-Tissue Expression (GTEx) database. Potential tumor antigens were screened by analyzing differentially expressed and mutated genes and potential antigens with significant differences in prognosis were screened using the Kaplan-Meier method. The proportion of immune cell infiltration in the tumor microenvironment was estimated using CIBERSORT and MCPcounter, and the correlation of potential antigens with antigen-presenting cells and major histocompatibility complex class II was analyzed. Subsequently, immune subtypes were constructed using consensus clustering analysis and characterized by single-sample gene set enrichment analysis and weighted gene co-expression network analysis (WGCNA). The Genomics of Drug Sensitivity in Cancer (GDSC) database was used to analyze the drug sensitivity of different immune subtypes.

**Results:** Four overexpressed and mutated tumor antigens associated with antigen presentation and poor prognosis were identified in ESCC, including NLRC5, FCRL4, TMEM229B, and LCP2. By consensus clustering, we identified two immune-associated ESCC subtypes, immune subtype 1 (IS1) and immune subtype 2 (IS2); the prognosis of the two subtypes was statistically different. In addition, the two immune subtypes had distinctly different cellular, molecular, and clinical characteristics. IS1 patients have a distinct immune “hot” phenotype with strong immune tolerance, whereas patients with IS2 have an immune “cold” phenotype. Differential expression of immune checkpoints and immunogenic cell death modulators was observed between the different immune subtypes. Finally, we found that IS1 and IS2 patients showed different drug sensitivities to common anti-tumor drugs, possibly facilitating the development of individualized treatment regimens for patients.

**Conclusion:** NLRC5, LCP2, TMEM229B, and FCRL4 are potential antigens for ESCC mRNA vaccines, and such vaccines may be more suitable for IS2 patients. This study provides a theoretical basis for mRNA vaccines against ESCC, by identifying the critical characteristics to predict ESCC prognosis and select suitable patients for vaccination.

## 1 Introduction

Esophageal cancer is one of the most aggressive and lethal malignant tumors of the human digestive system, ranking the sixth-highest of cancer-related deaths, with a 5-year survival rate of approximately 15–25%. (Huang and Yu, 2018; Sung et al., 2021) Esophageal cancer includes esophageal squamous cell carcinoma (ESCC) and esophageal adenocarcinoma, of which ESCC is the predominant pathological type. (Smyth et al., 2017) The region with the highest incidence of ESCC is East Asia, where ESCC accounts for more than 90% of esophageal cancers. (Pakzad et al., 2016) As localized ESCC is largely asymptomatic in its early stages, most patients are usually diagnosed at advanced or metastatic stages and therefore, cannot undergo surgical intervention. (Li et al., 2018) For patients with advanced ESCC, systematic treatment based on combination chemotherapy remains the main strategy. The efficacy of other treatments, such as molecular targeted drugs and immune checkpoint inhibitors, is not satisfactory due to off-target effects and immune resistance. (Ilson et al., 2011; Lu et al., 2016; Suntharalingam et al., 2017) Therefore, novel therapeutic strategies are needed to improve ESCC treatment.

Recently, the development of vaccines for cancer treatment has attracted significant attention from cancer researchers worldwide and may become a hot topic in cancer immunotherapy. (Sullenger and Nair, 2016; Pardi et al., 2018) Indeed, mRNA-based therapies were uncommon until the 2000s due to mRNA instability and the associated excessive inflammatory response. However, technological breakthroughs, including the development of effective delivery materials, optimization of coding sequences, and purification of IVT (*In Vitro* Transcription) mRNA have changed this by allowing mRNA to carry tumor antigens in their optimal form. (Gu et al., 2020; Pardi et al., 2020) Compared to traditional peptide vaccines, mRNA vaccines can be more easily modified to encode any protein, significantly improving vaccine productivity and shortening the therapeutic window for patients. Importantly, the half-life of mRNA can be adjusted by RNA sequence modifications or delivery systems to ensure safety. In addition, mRNA vaccines do not interfere with the host genome, thus avoiding insertional mutations. (Grunwitz and Kranz, 2017) To date, many studies have reported that mRNA vaccines encoding tumor-specific antigens promote anti-tumor immunity and prevent a variety of tumors, including melanoma, gastrointestinal tumors, lung cancer, pancreatic cancer, and hepatocellular carcinoma. (Cafri et al., 2020; Gu et al., 2020; Shahnazari et al., 2020; Khan et al., 2021) However, to date, no mRNA vaccine has been developed against the ESCC antigen, and no patients have been identified as suitable for vaccination.

The present study aims to explore neoantigens for the development of mRNA vaccines and to understand the immune landscape of ESCC to identify suitable patients for vaccination. Four candidate ESCC antigens were found to be associated with patient survival and antigen-presenting cell (APC) infiltration. Based on clustering analysis of immune-related genes, we defined two ESCC immune subtypes, finding that different immune subtypes corresponded to different clinical and immune characteristics. Finally, the immune characteristics of patients with different subtypes were analyzed to characterize the population eligible for mRNA vaccination. These findings point to a complex tumor immune microenvironment in ESCC patients and provide a theoretical basis for the development of mRNA vaccines and the selection of suitable vaccination populations.

## 2 Materials and Methods

### 2.1 Data Acquisition and Preprocessing

The RNA-seq data of ESCC and normal esophageal tissue were downloaded from the UCSC Xena (https://xenabrowser.net/) (Goldman et al., 2018). We used the “RNA-Seq by Expectation-Maximization (RSEM) expected_count” (*n* = 19,109) and “RSEM tpm” (*n* = 19,131) data from the “TCGA TARGET GTEx” dataset and extracted the transcriptome data of ESCC (*n* = 78) and normal esophageal tissue (*n* = 665).

The ESCC somatic mutation data were obtained from TCGA GDC (https://portal.gdc.cancer.gov/), selecting the “Masked Somatic Mutation” (*n* = 94), applying VarScan to preprocess the data, and using the maftools R package (Mayakonda et al., 2018) to visualize the somatic mutation landscape. The TMB of ESCC patients was calculated using “tmb” function of maftools package. The MSI score was obtained from previous published study. (Bonneville et al., 2017)

“Masked Copy Number Segment” (*n* = 95) was downloaded through the TCGAbiolinks R package (Colaprico et al., 2016), and the copy number variation (CNV) segment was analyzed by GISTIC 2.0 through GenePattern (https://cloud.genepattern.org) to study the CNV. (Reich et al., 2006).

Subsequently, the clinical phenotypes of patients (*n* = 95) matched by TCGA-ESCC were obtained using UCSC-Xena, including age, sex, survival information, and clinical staging.

### 2.2 Screening for Potential Tumor Antigens

#### 2.2.1 Gene Expression and Genetic Variation Analysis

Differential analysis between ESCC and normal esophageal tissues was performed using the DESeq2 R package (Love et al., 2014). Genes with *p*-values < 0.05 and absolute values of log_2_ (fold change) > 1 were considered as differentially expressed genes (DEGs). Among them, DEGs with log_2_ (fold change) > 1 were overexpressed genes. In addition, mutated genes and amplification of CNV categories were filtered based on the preprocessing data.

#### 2.2.2 Survival Analysis

We integrated the genes with overexpression and genetic variation, considering them to be potential antigens against ESCC. Then, Kaplan–Meier estimates with a median cutoff were performed to evaluate the overall survival (OS) of ESCC patients in whom potential antigens were identified. Significance was tested using the log-rank test, and a *p*-value < 0.05 was considered statistically significant.

#### 2.2.3 Correlation Analysis Between Potential Antigens and Infiltrated Immune Cells

The MCPcounter (Becht et al., 2016) and CIBERSORT (Newman et al., 2015) algorithms were used to evaluate the abundance of tumor immune infiltrating cells (TIICs) in the tumor samples. We then calculated the correlation between the expression of potential ESCC antigens and APCs and with several human leukocyte antigen (HLA) proteins from major histocompatibility complex (MHC) class II, commonly found in specialized APCs.

### 2.3 Identification of Immune Subtypes in ESCC

After gathering the immune-related gene set from the IMMPORT database (https://www.immport.org/shared/home) (Bhattacharya et al., 2018), the immune-related gene expression (TPM) was extracted from the TCGA-ESCC cohort. All tumor samples were clustered using the R package “ConsensusClusterPlus (Wilkerson and Hayes, 2010)”. The clustering algorithm used the k-means of Euclidean distance, the total number of subsampling was set to 50, and 80% of the total sample proportion was selected for each resampling. Cluster sets varied from 2 to 9, and the optimal partition was defined by evaluating the consensus matrix and the consensus cumulative distribution function. K value refers to the desired number of clusters, and the optimal K was determined using the elbow method, ensuring that the number of patients in each cluster was appropriate.

### 2.4 Immune Landscape and Prognostic Evaluation of Different Subtypes

The mRNA expression (TPM) and 28 immune signatures (Zheng et al., 2021), representing diverse immune functions, pathways, and immune cell types, were used for single-sample gene set enrichment analysis (ssGSEA) in the TCGA-ESCC cohort. The R package GSVA (Hänzelmann et al., 2013) was used to calculate the relative abundance enrichment score of each immune cell type, and the Mann–Whitney *U* test was used to compare the scores of different immune subtypes.

The log-rank test was used to compare the survival of different immune subtypes and to combine the clinical characteristics of each patient with ESCC, such as age, sex, stage, and survival status, to evaluate the proportion of ESCC patients with different immune subtypes.

### 2.5 Molecular and Immune Characteristics in Immune Subtypes

The ESTIMATE algorithm (Yoshihara et al., 2013) was used to quantify immune infiltration, stromal infiltration, and tumor purity. Immune cell death modulator (ICD)-and immune checkpoint (ICP)-related genes were obtained from previous studies. (Huang et al., 2021a; Huang et al., 2021b) The Mann–Whitney *U*-test was used to compare the molecular and immune characteristics between immune subtypes.

### 2.6 Weight Gene Co-Expression Network Analysis and Enrichment Analysis

The R package WGCNA was used to identify co-expression modules and explore the relationship between gene networks and immune subtypes. (Langfelder and Horvath, 2008) NormalizeBetweenArrays function was used for normalization of expression data. Then, the soft threshold was calculated using the pickSoftTreshold function, and a scale-free network was constructed based on the soft threshold. The minimum number of genes per module was set to 100. Subsequently, we calculated the dissimilarity of the module eigengenes and performed hierarchical clustering. Finally, we analyzed the correlation between modules and immune subtypes, selecting genes in the most correlated modules.

The predictive ability of each gene from the most correlated modules for prognosis was assessed using the receiver operating characteristic (ROC) curve plotted using the R package timeROC (Blanche et al., 2013); AUC >0.8 was considered a better predictive biomarker.

### 2.7 Immune Therapy Response and Drug Sensitivity of Immune Subtypes

The Genomics of Drug Sensitivity in Cancer (GDSC) database (www.cancerrxgene.org/) can be used to identify anti-tumor drugs and sensitive biomarkers. (Yang et al., 2013) The R package pRRophetic (Geeleher et al., 2014) was used to construct a ridge regression model to predict the sensitivity of different immune subtypes to common anti-cancer drugs based on IC50 values.

In addition, we used the tumor immune dysfunction and exclusion (TIDE) method (Jiang et al., 2018), which integrates the expression signatures of T-cell dysfunction and T-cell exclusion to model tumor immune evasion, to evaluate the predicted efficacy of immunotherapy in different immune subtypes.

## 3 Results

### 3.1 Identification of Potential ESCC Antigens

**FIGURE 1:**
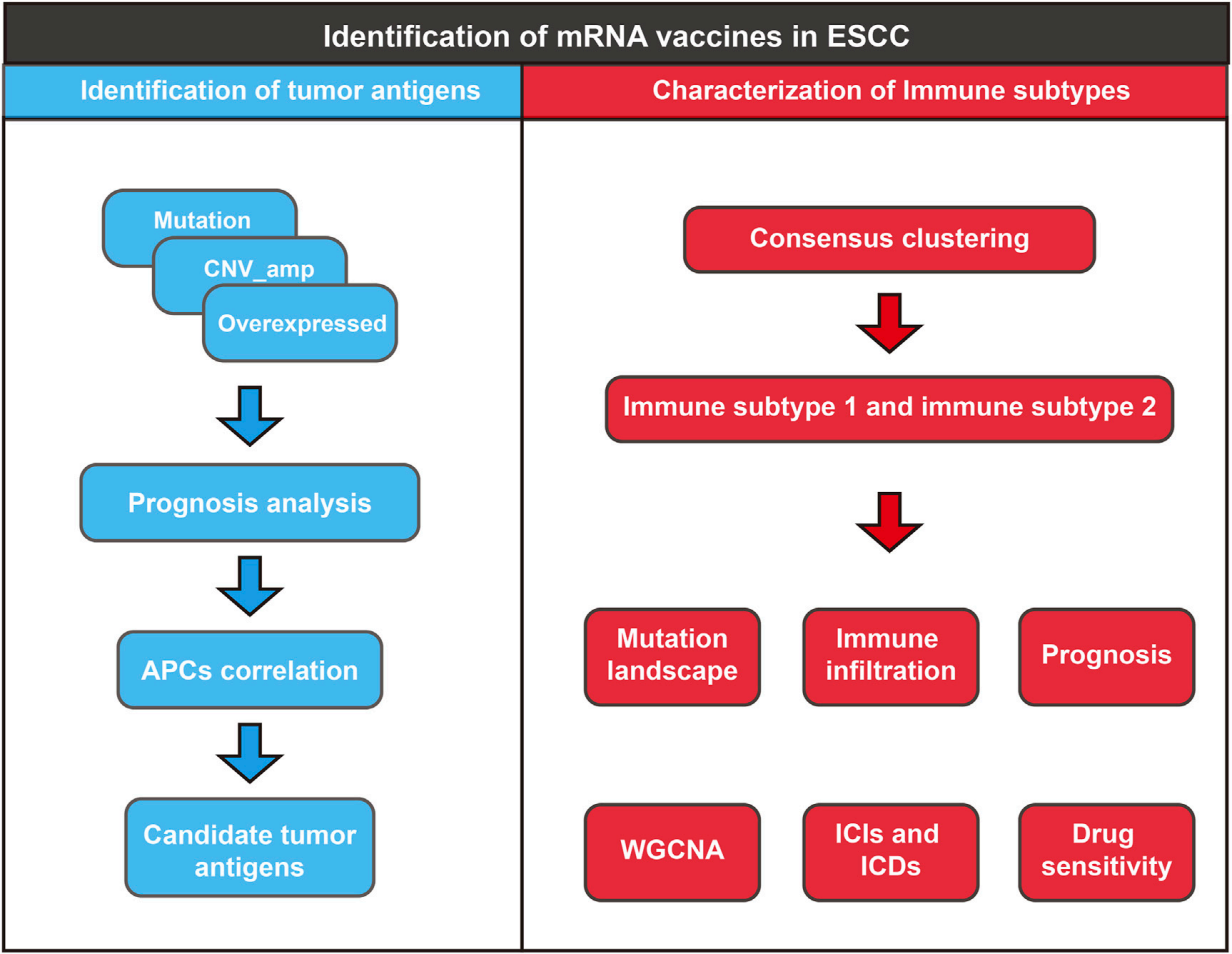
The work flow of this study.

**FIGURE 2:**
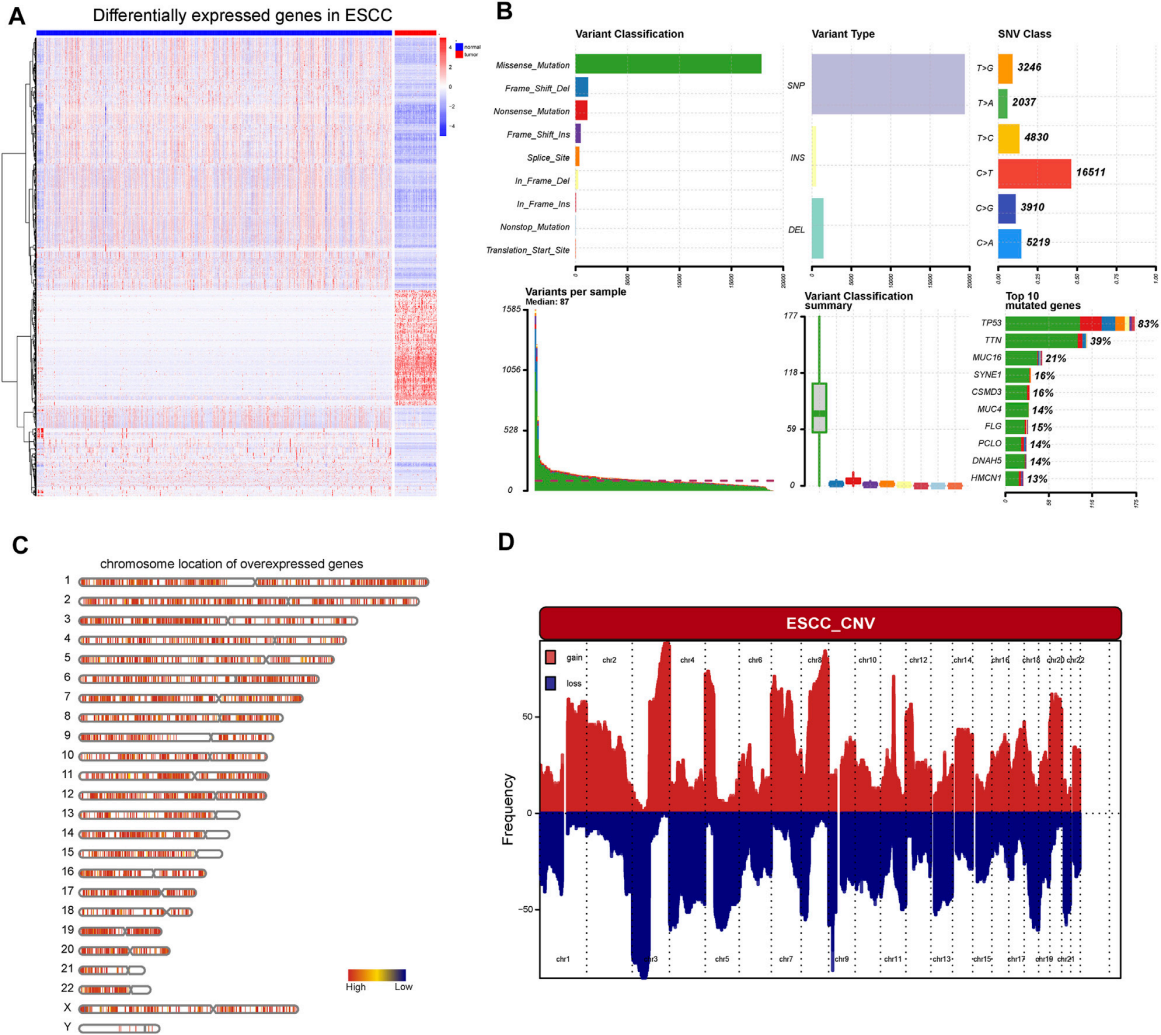
Identification of potential tumor antigens of ESCC. **(A)** Heatmap showing the DEGs in ESCC, including over-expressed genes and under-expressed genes. **(B)** The mutation landscape of ESCC, including the variant classification, variant type, SNV class, variate per sample, variant classification, and top 10 mutated genes. **(C)** Chromosomal distribution of over-expressed genes in ESCC **(D)** The aberrant copy number genes in ESCC.

First, the work flow of this study was shown in Figure 1. Aberrant gene expression, CNVs, and mutations caused by genomic instability are crucial contributors to cancer development. Therefore, to identify tumor-specific antigens in ESCC as potential mRNA vaccine targets, we first investigated aberrantly expressed genes. A total of 9,863 DEGs were detected in the comparison between ESCC tissues and normal tissues, including 5,836 overexpressed in ESCC tissues (Figures 2A,B). Then, we explored the overall mutational landscape of TCGA-ESCC. Missense and nonsense mutations occurred at higher frequencies, and a single nucleotide variant was the most frequent type of mutation (Figure 2C). Among them, 9,995 mutated genes, which potentially encode specific antigens, were screened in each ESCC patient. Subsequently, we used the GISTIC 2.0 software to investigate chromosomal variation and considered genes affected by regions with significant chromosomal amplification or deletion as potential mRNA vaccine targets (Figure 2D). A total of 3,824 genes with overexpression or variations in copy number and mutations were identified.

### 3.2 Identification of Tumor Antigens Associated With ESCC Prognosis and Antigen Presentation

**FIGURE 3:**
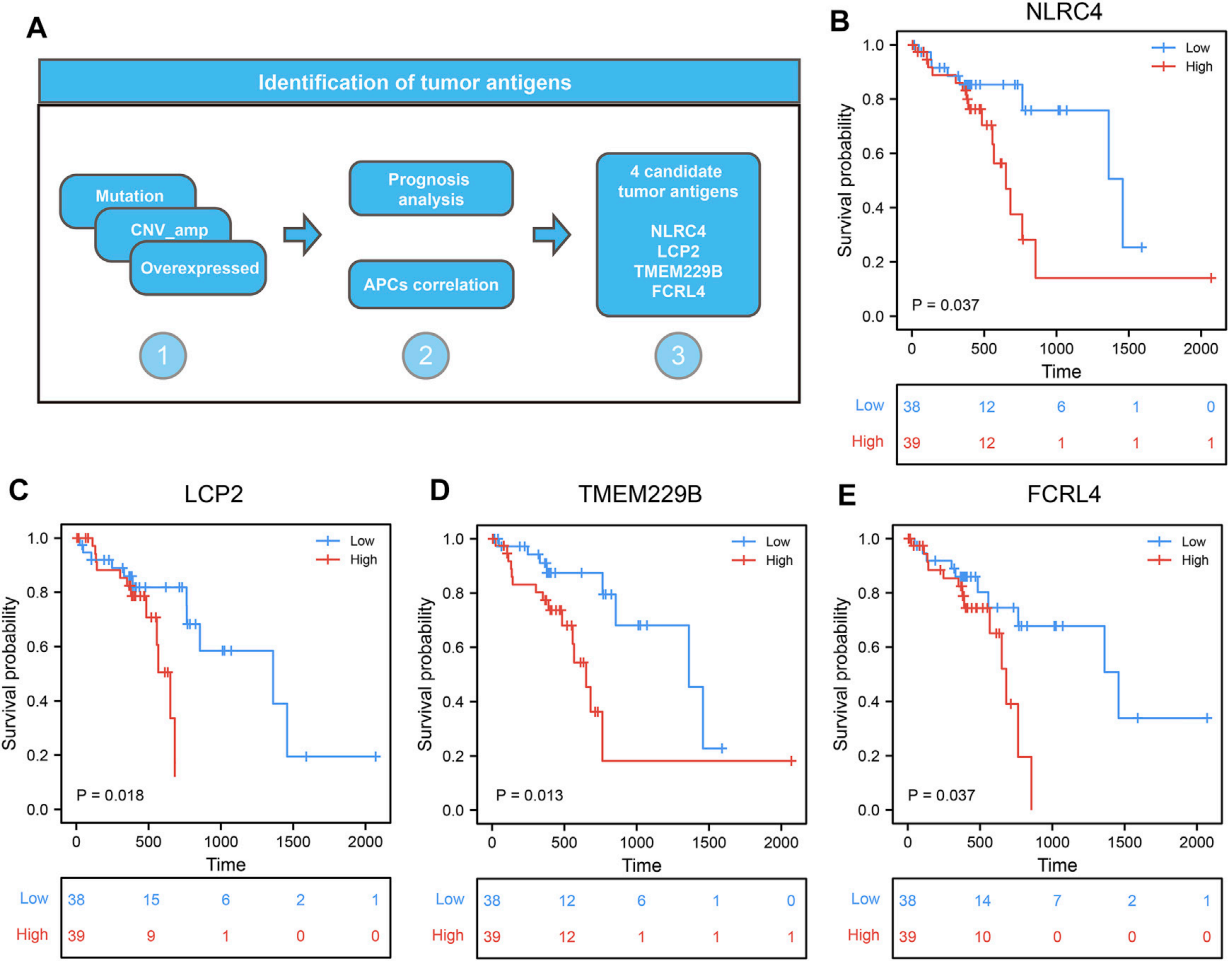
Identification of candidate tumor antigens associated with ESCC prognosis. **(A)** The procession of identifying candidate tumor antigens in ESCC. **(B–E)** Kaplan–Meier curves comparing the groups with different NLRC5 **(B)**, LCP2 **(C)**, TMEM229B **(D)**, and FCRL4 **(E)** in ESCC.

**FIGURE 4:**
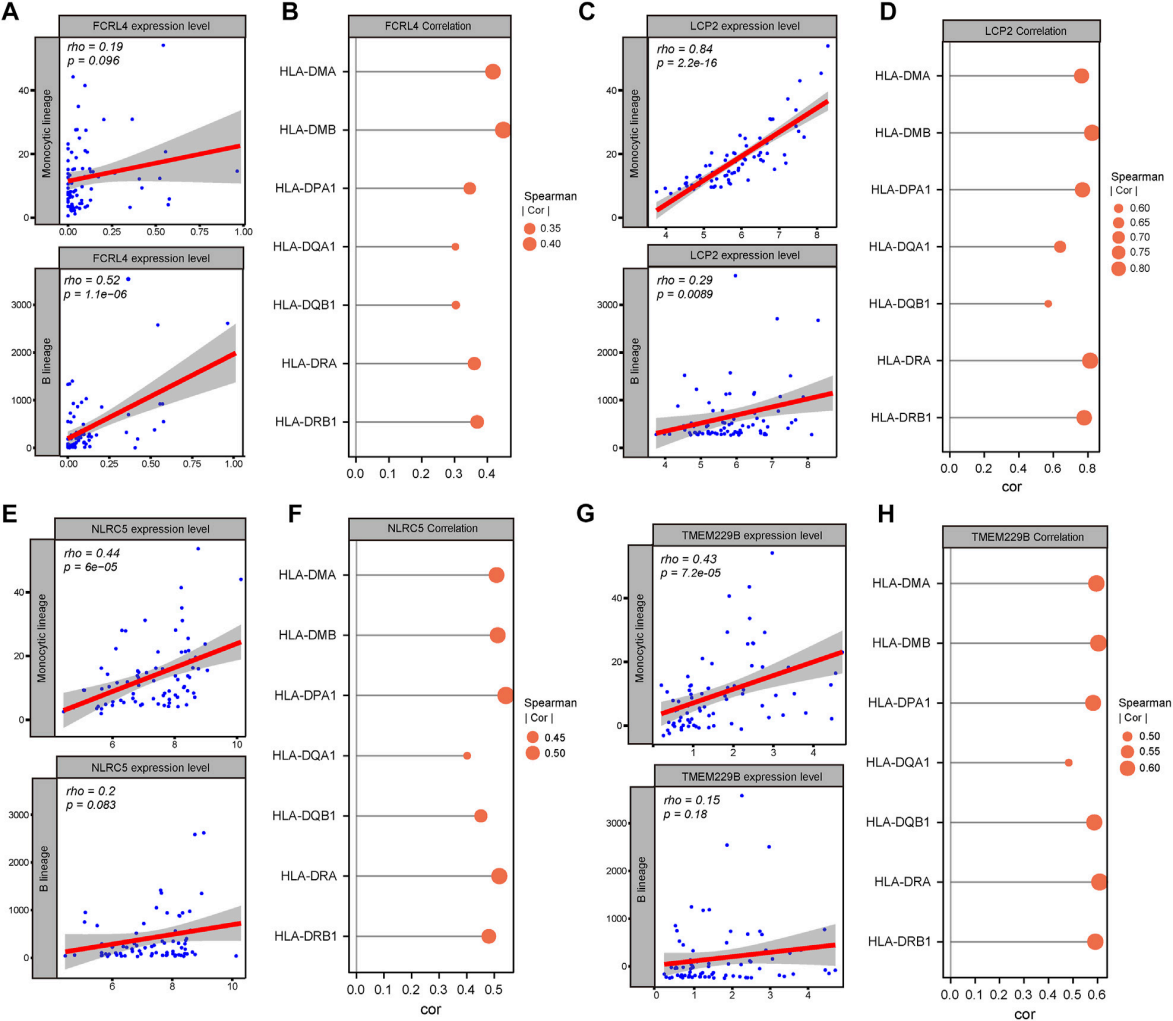
Identification of candidate tumor antigens associated with APCs and MHC class II. **(A)** Correlation between FCRL4 expression and estimated macrophage and B cell scores. **(B)** Correlation between FCRL4 expression and MHC II expression. **(C)** Correlation between LCP2 expression and estimated macrophages and B cells scores. **(D)** Correlation between LCP2 expression and MHC II expression. **(E)** Correlation between NLRC5 expression and estimated macrophages and B cells scores. **(F)** Correlation between NLRC5 expression and MHC II expression. **(G)** Correlation between TMEM229B expression and estimated macrophage and B cell scores. **(H)** Correlation between TMEM229B expression and MHC II expression.

To further identify the candidate tumor antigens associated with prognosis and antigen presentation, we performed a survival analysis of candidate antigens and correlation analysis between candidate antigens and APCs and HLA genes from MHC class II. After screening these genes, the NLR family CARD domain containing 5 (NLRC5), lymphocyte cytosolic protein 2 (LCP2), transmembrane protein 229B (TMEM229B), Fc receptor like 4 (FCRL4) were eligible (Figure 3A). These four genes were highly expressed, and mutated sites were detected in ESCC tissues (Supplementary Figures S1A–H). We also found that ESCC patients with high expression levels of these four genes were associated with poor prognosis (Figures 3B–E). These results suggested that these four genes might play significant roles in the initiation and progression of ESCC. Moreover, the elevated expression levels of these four genes, such as B cells and macrophages, was associated with increased infiltration of APCs (Figures 4A,C,E,G). Notably, the correlation between these four genes with MHC class II genes, like *HLA-DMA, HLA-DMB, HLA-DPA1*, etc. were clear positive (Figures 4B,D,F,H). Therefore, these four tumor antigens (NLCR4, LCP2, TMEM229B, FCRL4) were considered as potential candidates for ESCC mRNA vaccine with immune activation and can be processed and presented by APCs to induce an immune response.

### 3.3 Identification of Potential ESCC Immune Subtypes

**FIGURE 5:**
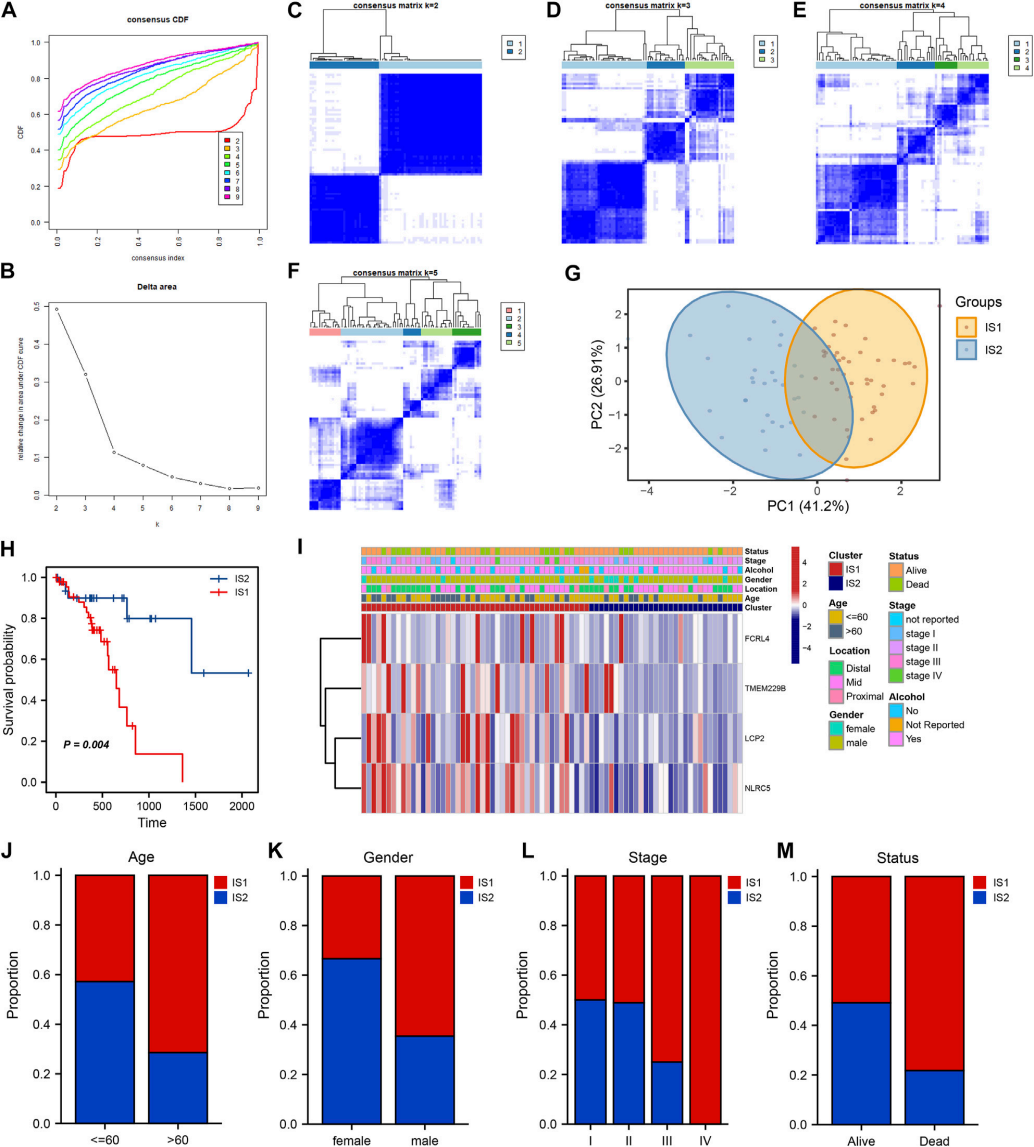
Identification of potential ESCC immune subtypes. **(A)** Consensus clustering the cumulative distribution function curve for k = 2 to k = 9. **(B)** Relative change in area under the cumulative distribution function curve. **(C–F)** Clustering heatmap for k = 2 to k = 5. **(G)** PCA plot showing the distribution of IS1 and IS2. **(H)** Kaplan–Meier curve showing the OS of IS1 and IS2 in the TCGA cohort. **(I)** Heatmaps showing the relationship between potential ESCC antigens and clinical characteristics in different immune subtypes. **(J–M)** Distribution ratio of IS1 and IS2 across age, gender, stage and survival status in ESCC patients.

Understanding the immune subtypes can help us understand the tumor microenvironment and immune status, identify different patients, and assess their suitability for mRNA vaccination. An expression profile, containing 2,483 immune-related genes extracted from TCGA-ESCC, was implemented for consensus clustering to identify different immune subtypes of ESCC patients. The tracking plot and clustering analysis results showed that the optimal number of clusters was two, with sample numbers of 46 and 31, respectively (Figures 5A–F). The cluster containing more samples was defined as immune subtype 1 (IS1), and the other was defined as immune subtype 2 (IS2). Principal component analysis (PCA) revealed separation between the two clusters (Figure 5G). The survival analysis indicated that IS2 was associated with better survival probability, whereas IS1 had a poorer prognosis (Figure 5H). Notably, four candidate tumor antigens showed a consistent expression trend, in which their expression levels were decreased in the IS2 (Figure 5I). Moreover, several clinical characteristics, such as age, sex, stage, and status, displayed different distributions in the two subtypes. For example, a higher IS1 proportion was observed in patients older than 65 years and in advanced stages. In addition, the patients in IS1 account for a large portion in the “Dead” status (Figure 5J–M).

### 3.4 Association of Immune Subtypes With Mutational Status

**FIGURE 6:**
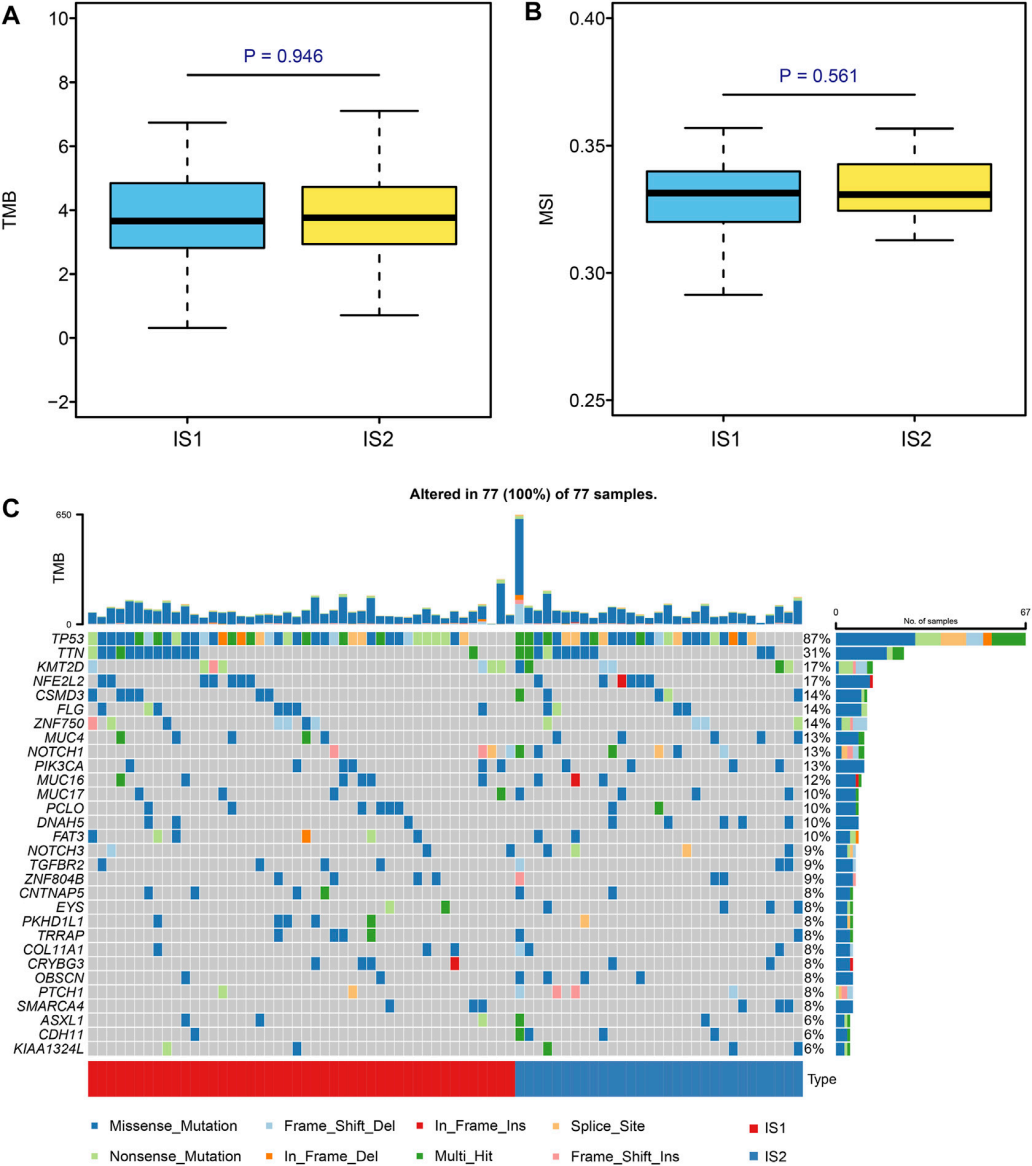
Identification of mutation signature in different subtypes. **(A,B)** Comparison of TMB and MSI in ESCC patients with different immune subtypes. **(C)** Top 30 mutated genes in the IS1 and IS2 subtypes.

Microsatellite instability-high (MSI-H) and high tumor mutation burden (TMB) are correlated with stronger anti-cancer immunity. (Rooney et al., 2015) First, we viewed the ESCC patient mutation landscape of IS1 and IS2. As shown in Supplementary Figures S2A, B, there were no detectable differences between IS1 and IS2 in variant classification, variant type, SNV class, etc. We noticed that the top 10 mutated genes in IS1 were *TP53, TTN, MUC16, ZNF750, NFE2L2, KMT2D, FLG, PCLO, FAT3*, and *CSMD3*, whereas the top 10 mutated genes in IS2 were *TP53, TTN, NOTCH1, KMT2D, CSMD3, NFE2L2*, *CDH11, PAPPA2, MUC4*, and *DNAH5*. Then, we calculated the TMB and MSI of IS1 and IS2, finding no statistically significant difference between the two clusters (Figures 6A,B). Moreover, we extracted the top 30 mutated genes in IS1 and IS2 and visualized them using the maftools R package (Figure 6C). There was no significant difference in the immune signatures between IS1 and IS2. However, several genes with different mutated frequencies might help distinguish immune subtypes. Thus, we thought that patients should be evaluated, and, for example, target gene sequencing should be performed before considering vaccination.

### 3.5 Characteristics of the Tumor Immune Microenvironment in Various Immune Clusters

**FIGURE 7:**
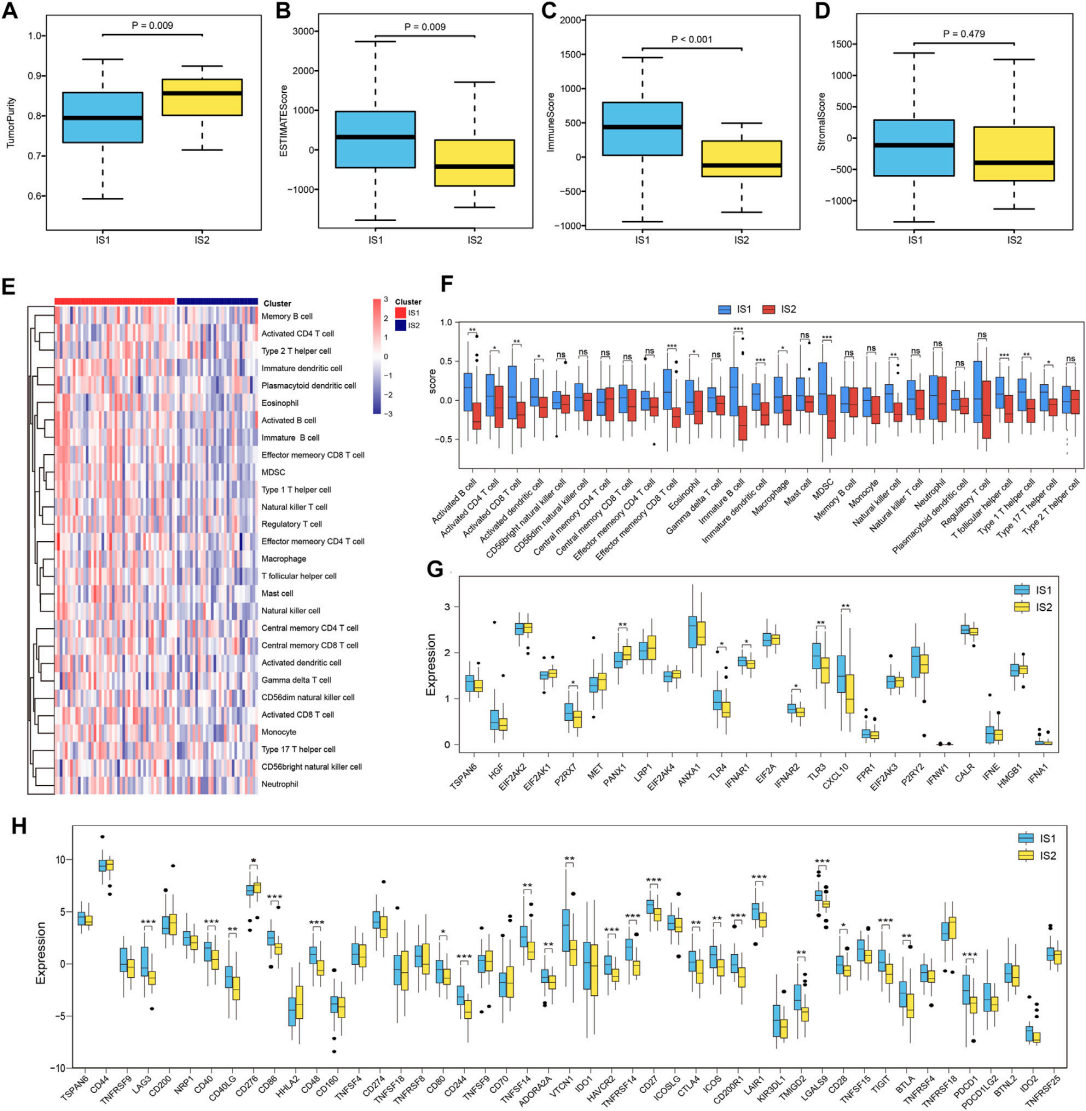
Immune-related molecular characteristics in different subtypes. **(A–D)** Comparison of tumor purity, estimate score, immune score, and stromal score based on the ESTIMATE algorithm with different ESCC patient subtypes. **(E)** Heatmap showing the 28 immune cell signatures with differential enrichment score in IS1 and IS2 subtypes. **(F)** Boxplot showing the differential enrichment score according to the 28 immune cell signatures in IS1 and IS2 subtypes. **(G)** Boxplot showing the differential expression of ICPs in the IS1 and IS2 subtypes. **(H)** Boxplot showing the differential expression of ICDs in IS1 and IS2 subtypes.

The immune status of the tumor microenvironment affects the response of the mRNA vaccine. Thus, we further assessed immune and stromal scores using the ESTIMATE algorithm to estimate the immune status of the tumor microenvironment of each immune subtype. Upon comparison, we found that IS1 had a higher immune and estimate scores than IS2, whereas tumor purity in IS1 was lower than that in IS2 (Figures 7A–D). Moreover, the evaluation of immune cell infiltration calculated using ssGSEA showed that the composition of TIICs was significantly different among the immune subtypes (Figure 7E). Many TIICs showing a consistent trend that TIICs in IS1 exhibited higher infiltration scores than IS2, like activated B cells, activated CD4 T cells, activated CD8 T cells, activated dendritic cells, etc. (Figure 7F). Therefore, IS1 can be considered “hot,” with high immune infiltrate, whereas IS2 was “cold,” with low immune infiltrate. These results may suggest that IS1 patients respond better to immunotherapy and mRNA vaccines, while IS2 patients respond less well to immune-related treatments.

Furthermore, we compared the expression levels of ICPs and ICD modulators in different immune subtypes and found that the overall expression levels of ICPs and ICDs in IS1 were higher than those in IS2. Among these biomarkers, we found that PDCD1, CTLA-4, CD40, and CD48 were overexpressed in IS1 (Figures 7G,H). These ICPs and ICD modulators can serve as promising biomarkers for mRNA vaccines.

### 3.6 Identification of Immune Gene Co-Expression Module in ESCC

**FIGURE 8:**
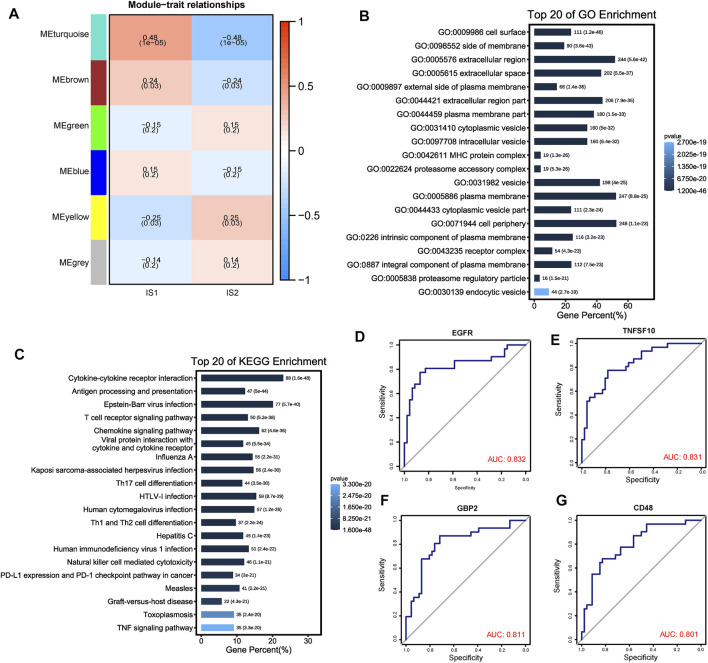
Identification of immune gene co-expression modules of ESCC. **(A)** Heatmap showing the relationship between the gene module eigenvalues and different immune subtypes. The number above each model represent correlations, numbers below represent *p* value. **(B, C)** Top 20 GO and KEGG enriched functions and pathways of “MEturquoise” module genes. **(D–G)** ROC plots showed the four genes with a suitable predictive ability for the prognosis with AUC >0.8.

ESCC samples were clustered using the WGCNA algorithm, and “four” was chosen as the soft threshold power based on the scale-free fit index and average connectivity (Supplementary Figures S3A, B). The representation matrix was then converted to an adjacency matrix and then to a topological matrix. We used the average-linkage hierarchy clustering method with a minimum of 100 genes for each network, following the hybrid dynamic shear tree standard. Six modules were identified among all the immune-related genes (Figure 8A). After further investigating the association between modules and phenotypes, we observed that the “turquoise” module was the characteristic module of IS1, suitable for vaccination. After extracting the genes from the “turquoise” module, enrichment analysis results showed that the genes were involved in multiple immune-related functions, such as antigen processing and presentation, T-cell receptor signaling pathway, Th1- and Th2-cell differentiation, PD-L1 expression, and PD-1 checkpoint pathway in cancer (Figures 8B,C). Moreover, we further investigated the predictive ability of genes from the “turquoise” module for the prognosis in the IS1 population and identified four genes (*EGFR, TNSF10, GBP2,* and *CD48*) with suitable prognostic features, using ROC curves AUC >0.8 as a screen (Figures 8D–G). Therefore, these four genes could serve as promising biomarkers for mRNA vaccination.

### 3.7 Estimation of Correlation Between Immune Subtypes and Anti-Cancer Drug Sensitivity

**FIGURE 9:**
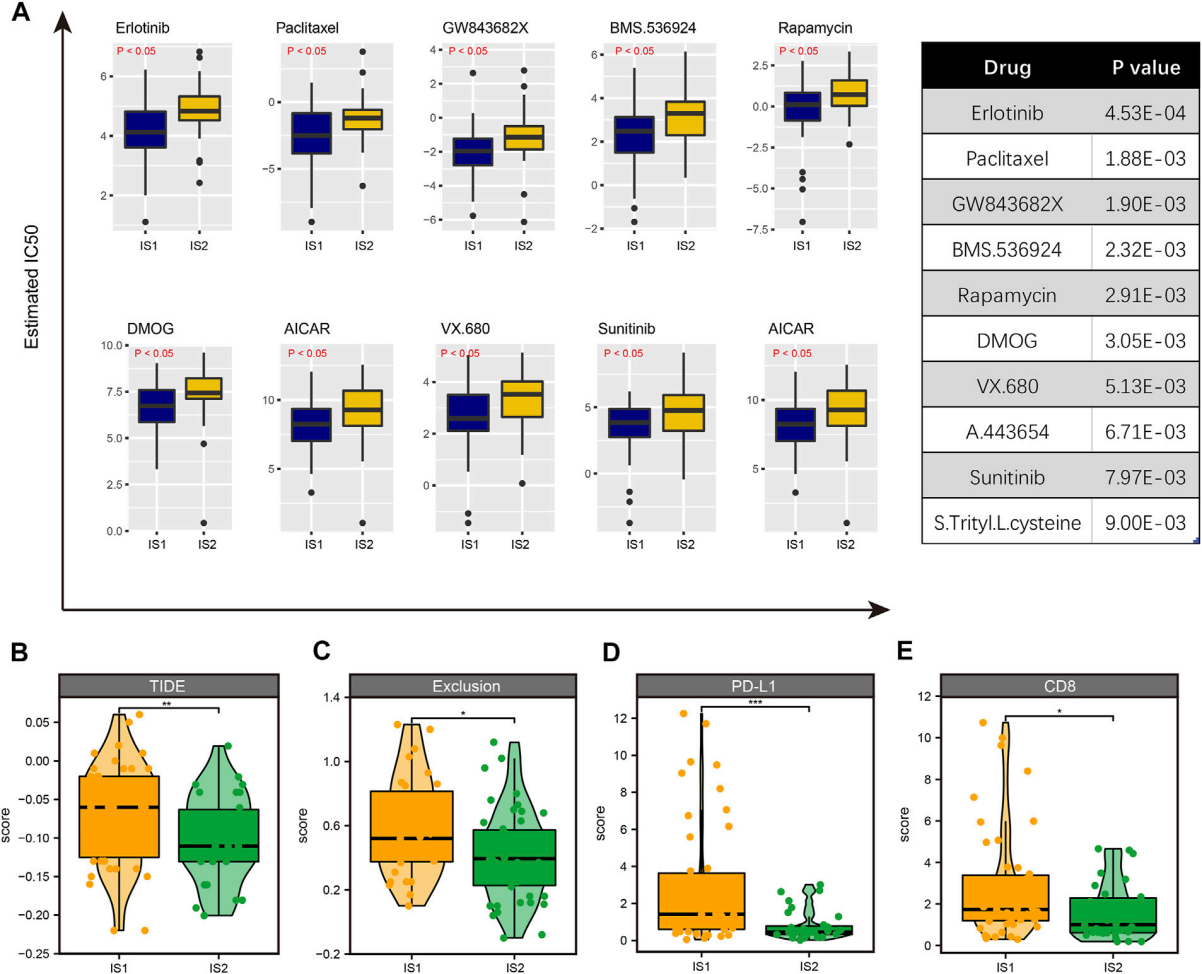
Identification of drug sensitivity in IS1 and IS2 subtypes. **(A)** The 10 drugs with the most significant differences ranked by *p*-value. **(B)** The TIDE score is higher in IS1 than in IS2. **(C)** The exclusive score is higher in IS1 than in IS2. **(D)** The PD-L1 score is higher in IS1 than in IS2. **(E)** The CD8 score is higher in IS1 than in IS2.

Although we have performed immunophenotyping on patients with ESCC and explored the differences in the sensitivity of mRNA vaccines to patients with different subtypes, there are still some patients who are not sensitive to mRNA vaccines that cannot benefit from it. Hence, we used drug sensitivity data from the GDSC database as a training set to predict common anti-cancer drug sensitivity in TCGA-ESCC samples. A total of 138 common drugs were included in the analysis. After comparison, we found significant sensitivity differences between multiple drugs in IS1 and IS2 species, such as Erlotinib, Paclitaxel, GW843682X, BMS.536,924, Rapamycin, DMOG, etc. The ten drugs with the most significant differences ranked by *p*-value were shown in Figure 9A. Interestingly, we found that all these drugs were more sensitive in IS1 than IS2. Then, we further estimated the sensitivity of different immune subtypes to immunotherapy using the TIDE database, finding that TIDE scores were higher in IS1 than IS2; other immunotherapy marker scores, such as CD8 and CD274, were higher in IS1 (Figures 9B–E). Thus, patients in IS2 might be suitable for mRNA vaccination and immunotherapy, and patients with IS1 might be more sensitive to targeted therapies. The difference in treatment sensitivity between immune subtypes also reflects the heterogeneity of tumors, further emphasizing the importance of individualized treatment.

## 4 Discussion

Currently, mRNA technology is one of the main modalities for tumor vaccines under development. Neoantigens can be identified by exome sequencing the patient population and comparing the sequencing data with the data from healthy individuals. The mRNA vaccines based on the obtained neoantigen sequences can subsequently be delivered to specific cells to translate specific antigens. The body’s immune system recognizes these neoantigens that share the same antigenic determinant clusters as the surface antigens of disease-causing pathogens and tumors, thereby eliciting immune response. (Zhu et al., 2017; Sang et al., 2019; Asselah et al., 2021) To date, several studies have reported the application of mRNA vaccines in tumor therapy, offering a broad therapeutic prospect for the individualized treatment of tumor patients. (Persano et al., 2017; Liu et al., 2018; Wang et al., 2018; Cafri et al., 2020)

Surgical resection is an important treatment modality for patients with early stage resectable ESCC. For patients with advanced stage disease that cannot be treated surgically, chemotherapy is currently the main modality—often a combination of targeted therapy and immunotherapy is used. (Chen et al., 2019; Wang et al., 2021) The potential utility of mRNA cancer vaccines in patients with ESCC has not been explored, leaving the clinical benefit unclear. In the present study, we constructed an ESCC aberrant expression and genomic alteration landscape to reveal a broad range of potent antigenic targets that may be considered to treat ESCC. As neoantigens predicted using aberrant expression and genetic alteration profiles may not be functionally significant in ESCC, prognostic analysis and immune correlations were further used to confirm the clinical relevance of the selected antigens. Candidate ESCC antigens, NLRC5, LCP2, TMEM229B, and FCRL4, were selected after screening; upregulation of the antigens is associated with poor prognosis, high infiltration of APCs, and high level of MHC class II gene expression. These antigens might play a significant role in the development and progression of ESCC and can be presented directly to CD8^+^ T-cells to induce an immune response in the presence of adequate lymphocyte infiltration. Interestingly, we noticed that these candidates have been explored in several previous studies. For instance, NLRC5 is upregulated in some tumors and has been proven to play a significant role in cancer immune surveillance through the recruitment and activation of CD8^+^ T-cells. (Yoshihama et al., 2017; Tang et al., 2020) Furthermore, LCP2 was overexpressed in lung adenocarcinoma, diffuse large B-cell lymphoma, and melanoma, and it was associated with poor prognosis. (Long et al., 2021; Pan et al., 2021; Wang and Peng, 2021) However, studies on TMEM229b and FCRL4 in tumors are scarce, and the functions they play in tumors remain unknown. Considering that the therapeutic response and survival benefit in patients receiving tumor-based vaccine therapy remains limited to a small subset of patients, we further identified the patient subtype according to tumor immune-related gene profiles to provide guidance on the optimal use of tumor vaccine therapy. Different gene expression profiles and clinical prognoses helped characterize two immune subtypes. The two immune subtypes exhibit different molecular, immunological, and clinical characteristics. There was a significant difference between the prognosis of IS1 and IS2 patients, with the prognosis of IS2 patients being better than that of IS1 patients. In addition to prognosis, we compared the proportions of the two subtypes by age, sex, stage, and survival, finding that IS1 patients accounted for a greater proportion of elderly patients and patients with advanced ESCC. When we compared the mutational characteristics of IS1 and IS2 patients, we found that TMB and MSI were not clearly distinguishable between the two groups and that the differences in mutation profiles between the two groups were not significant; thus, we may not be able to distinguish between the two immune subtypes in terms of mutation profiles. Moreover, we analyzed the relationship between the two immune subtypes with immune cell infiltration and immune-related biomarkers. Higher immune cell infiltration was observed in IS1 patients than in IS2 patients, and the expression levels of most ICIs and ICDs was elevated in IS1 patients, suggesting that IS1 patients may be more suitable for mRNA vaccines. Subsequently, based on the GDSC and TIDE databases, we further analyzed the therapeutic response of other treatment modalities, such as chemotherapy, targeted therapy, and immunotherapy, in IS1 patients and IS2 and developed a strategy consistent with individualized treatment accordingly.

In previous studies, Liang et al. screened potential tumor antigens in cholangiocarcinoma and pancreatic adenocarcinoma by bioinformatics and classified tumor patients into different subtypes by consensus clustering analysis to identify patients suitable for mRNA vaccination. (Huang et al., 2021a; Huang et al., 2021b) Subsequently, Tian et al. and Li et al. successively explored the feasibility of mRNA vaccines in glioma, providing a theoretical basis for the development of mRNA vaccines in glioma. (Ye et al., 2021; Zhong et al., 2021) Therefore, with the progress and development of sequencing technology, bioinformatics technology can be applied to find new directions for the development of mRNA tumor vaccines, characterization of suitable tumor antigens from a multi-omics perspective, and identification of suitable patients according to their immune subtype for vaccination, yielding new strategies for precise treatments.

In conclusion, NLRC5, LCP2, TMEM229B, and FCRL4 were determined to be potential antigens for ESCC mRNA vaccines, possibly more suitable for IS2 patients. This study provides a theoretical basis for developing mRNA vaccines against ESCC—it may facilitate accurate patient prognosis and select subsets of patients that may derive therapeutic benefit from such vaccination.

## Data Availability

Publicly available datasets were analyzed in this study. This data can be found here: TCGA (https://portal.gdc.cancer.gov/) and GTEx (https://www.genome.gov/Funded-Programs-Projects/Genotype-Tissue-Expression381 Project) databases.
