# Supplementary material for: Identification of Tumor Antigens and Immune Subtypes of Esophageal Squamous Cell Carcinoma for mRNA Vaccine Development
Source: Front Genet. 2022 Jun 6;13:853113. doi: 10.3389/fgene.2022.853113 (PMC9207414; doi:10.3389/fgene.2022.853113)
Supplement: Supplementary file 1 [file DataSheet1.PDF]

Supplementary information

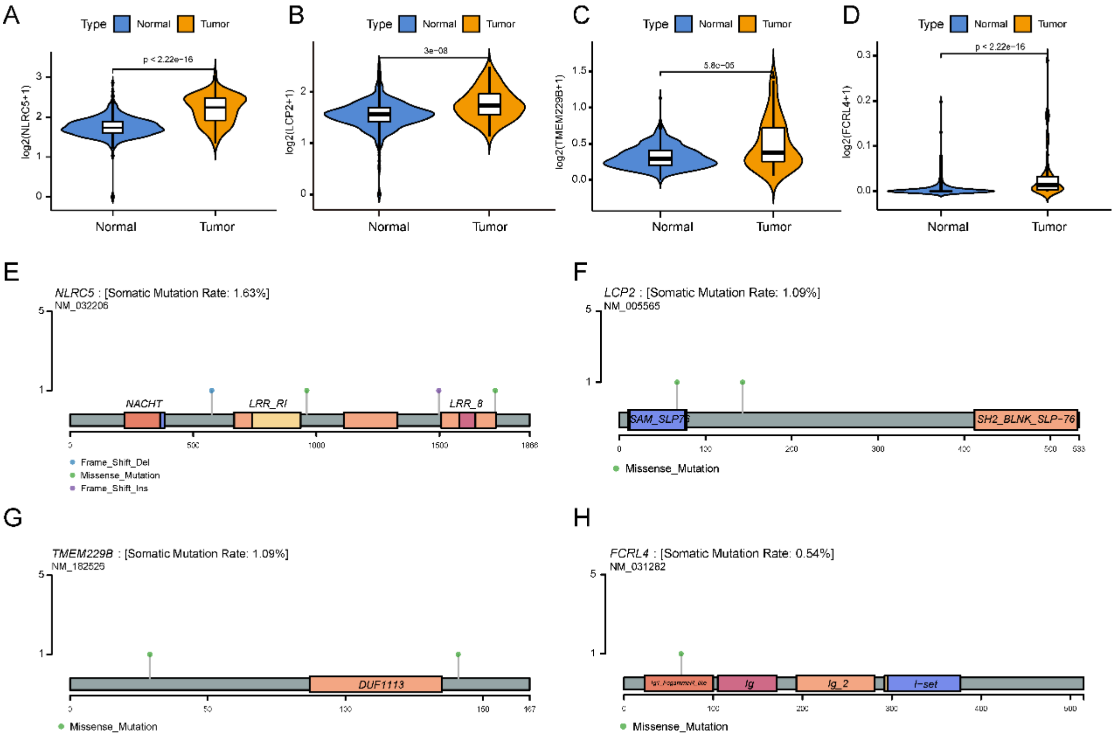

Figure S1. Expression and mutation information of candidate antigens. (A-D) NLRC5, LCP2, TMEM229B, and FCRL4 are overexpressed in ESCC tissues. (E–H) Mutated sites of NLRC5, LCP2, TMEM229B, and FCRL4.

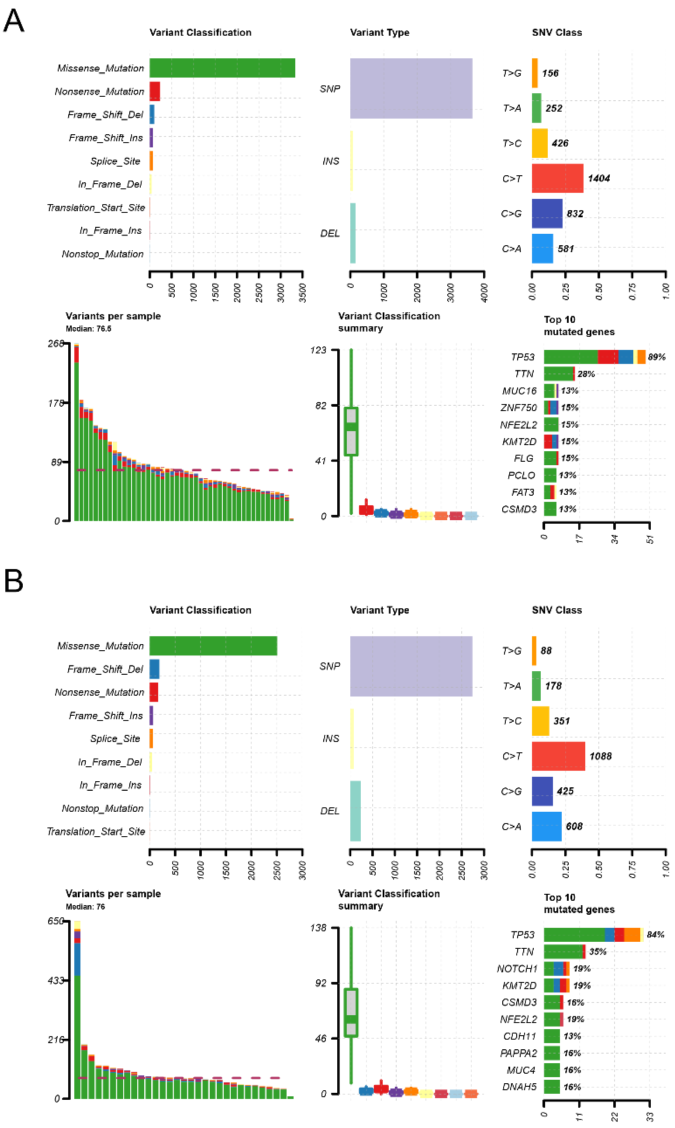

Figure S2. Immune landscape of IS1 and IS2. (A) Overall mutant landscape of IS1 patients. (B) Overall mutant landscape of IS2 patients.

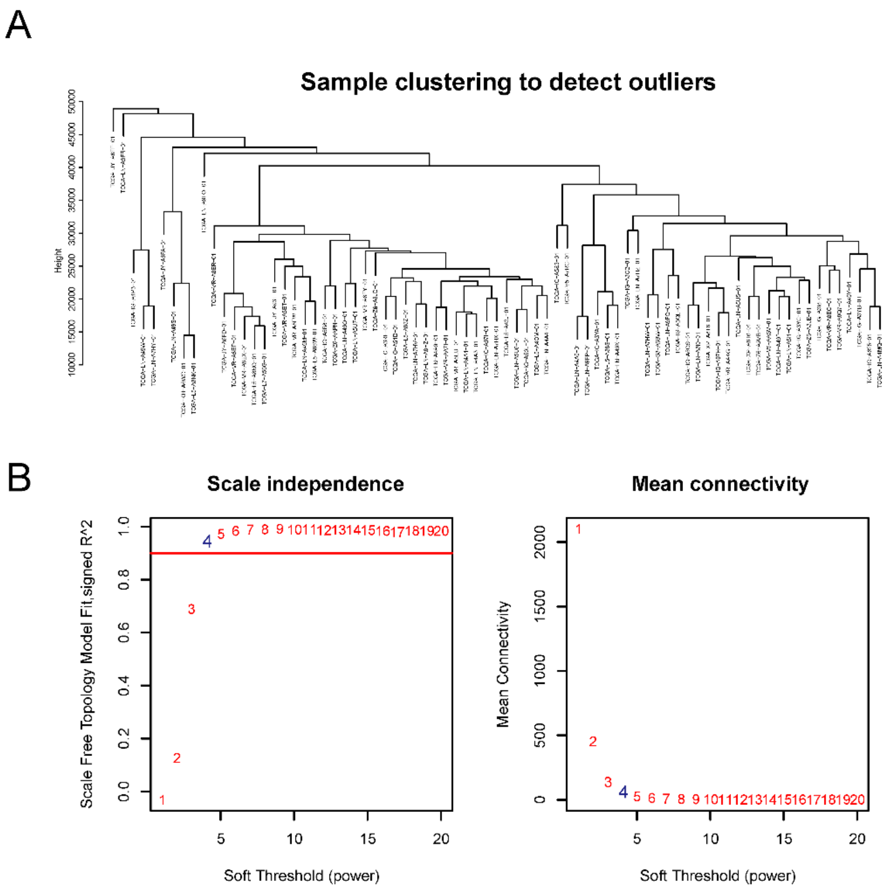

Figure S3. Quality control of WGCNA. (A) Sample clustering of ESCC patients in the TCGA-ESCC cohort. (B) Identification of best soft-thresholding power according to the scale-free fit index.
